# Recombination between Vaccine and Field Strains of Porcine Reproductive and Respiratory Syndrome Virus

**DOI:** 10.3201/eid2512.191111

**Published:** 2019-12

**Authors:** Anping Wang, Qi Chen, Leyi Wang, Darin Madson, Karen Harmon, Phillip Gauger, Jianqiang Zhang, Ganwu Li

**Affiliations:** Jiangsu Agri-animal Husbandry Vocational College, Taizhou, China (A. Wang);; Iowa State University, Ames, Iowa, USA (A. Wang, Q. Chen, D. Madson, K. Harmon, P. Gauger, J. Zhang, G. Li);; University of Illinois, Urbana, Illinois, USA (L. Wang);; Chinese Academy of Agricultural Sciences, Harbin, China (G. Li)

**Keywords:** PRRSV, recombination, vaccine, wild type, porcine reproductive and respiratory syndrome virus, viruses, pigs, swine, United States

## Abstract

We isolated and plaque purified IA76950-WT and IA70388-R, 2 porcine reproductive and respiratory syndrome viruses from pigs in the same herd in Iowa, USA, that exhibited coughing and had interstitial pneumonia. Phylogenetic and molecular evolutionary analysis indicated that IA70388-R is a natural recombinant from Fostera PRRSV vaccine and field strain IA76950-WT.

Porcine reproductive and respiratory syndrome (PRRS), characterized by reproductive failure in sows and respiratory distress in pigs of all ages, causes substantial economic loss to the worldwide swine industry. PRRS virus (PRRSV) is an enveloped, single-stranded, and positive-sense RNA virus belonging to the family *Arteriviridae* (*1*). Historically, PRRSV comprises type 1 (PRRSV-1) and type 2 (PRRSV-2); recently, PRRSV-1 was taxonomically classified into the species *Betaarterivirus suid 1* and PRRSV-2 into the species *Betaarterivirus suid 2*. PRRS has remained the most important disease of swine throughout the world, and live attenuated vaccines are used to reduce the clinical impact of PRRSV infection. Several studies have reported that recombinant PRRSV strains emerged in China, Korea, and France because of recombination between wild-type and vaccine strains (*2**–**6*). Nevertheless, recombination between a live attenuated vaccine strain and a circulating strain has not been reported in the United States.

In October 2018, a farm with a history of using Fostera PRRSV vaccine had been experiencing an ongoing problem with porcine respiratory disease. Histopathologic examination of 2 samples (lungs A and B) revealed the lungs of both pigs demonstrated significant interstitial pneumonia. Open reading frame (ORF) 5 Sanger sequencing identified a wild-type PRRSV from sample A and a vaccine Fostera-like PRRSV from sample B. However, the Fostera-specific real-time PCR, which targets the nonstructural protein (NSP) 2 region in the virus, was consistently negative for both samples. The viruses were isolated, plaque-purified, and sequenced on the Illumina MiSeq platform (Illumina, https://www.illumina.com) (Appendix). The 2 plaque-purified PRRSV isolates, IA76950-WT from pig A and IA70388-R from pig B, had 100% nt identities to those directly sequenced from the lung tissues.

We determined 14,980 and 14,987 nt of the full-length genomes of IA76950-WT (GenBank accession no. MK796164) and IA70388-R (GenBank accession no. MK796165). The whole genomes of IA76950-WT and IA70388-R shared 81.5% and 85.4% nt identity with the PRRSV-2 prototype strain VR-2332 but only 60.7% and 60.8% with the PRRSV-1 representative Lelystad strain, indicating that both isolates belonged to PRRSV-2. To evaluate the genomic characteristics of IA76950-WT and IA70388-R, we compared their genomes with all PRRSV-2 strains in GenBank and 12 representative strains, including NADC30, CH-1a, SDSU73, VR-2332, and selected 5 US vaccine strains for further analysis in detail (Appendix Table). IA70388-R had >99% nt identity to IA76950-WT in Nsp1α, Nsp1β, and Nsp2~5 and demonstrated much lower nucleotide identities (74.8%–89.8%) in the 3′ region encoding from Nsp6 to ORF7. In contrast, IA70388-R showed high nucleotide identities (99.3%–100%) to the Fostera PRRSV vaccine strain in Nsp6 to ORF7 and lower nucleotide identities in Nsp1α, Nsp1β, and Nsp2~5. These results suggested that IA70388-R might be a recombinant that evolved from IA76950-WT and the Fostera vaccine virus.

We further constructed a phylogenetic tree of the NSP2 gene, ORF5 gene, and whole-genome sequences using 12 representative field strains and 5 vaccine strains (Appendix Figure 1). IA76950-WT, IA70388-R, and Fostera vaccine strains were located in 3 different lineages based on the whole-genome sequences. For analysis of NSP2 sequence, IA76950-WT and IA70388-R formed a minor branch and clustered close to the MN184A and NADC30 but remotely from the lineages formed by Fostera, SDSU73, VR2332, and Ingelvac MLV. In contrast, the ORF5 sequence-based phylogenetic tree showed that IA70388-R clustered with Fostera vaccine strain in lineage L8, and the IA76950-WT clustered with NADC30, MN184, and Prevacent vaccine strains in lineage L1 (Appendix Figure 1). These results also suggested that IA70388-R might be a mosaic.

Finally, we aligned the complete genomes of IA76950-WT, IA70388-R, and the Fostera strains using ClustalX (http://www.clustal.org) and conducted a similarity plot analysis using SimPlot software (*7*). One recombination breakpoint was identified in the Nsp5 (nucleotide position 6742) separating the genome into 2 regions (Appendix Figure 2). IA70388-R was highly similar to that of IA76950-WT in the 5′ region with 99%–99.8% nt identities; however, IA70388-R had high similarity with the Fostera vaccine strain in the 3′ region with 99.3%–100% nt identities (Appendix Figure 2). In addition, we used RDP version 4.24 (http://web.cbio.uct.ac.za/~darren/rdp.html) to evaluate potential recombinants, and it completely confirmed the results of SimPlot analysis (Figure).

**Figure:**
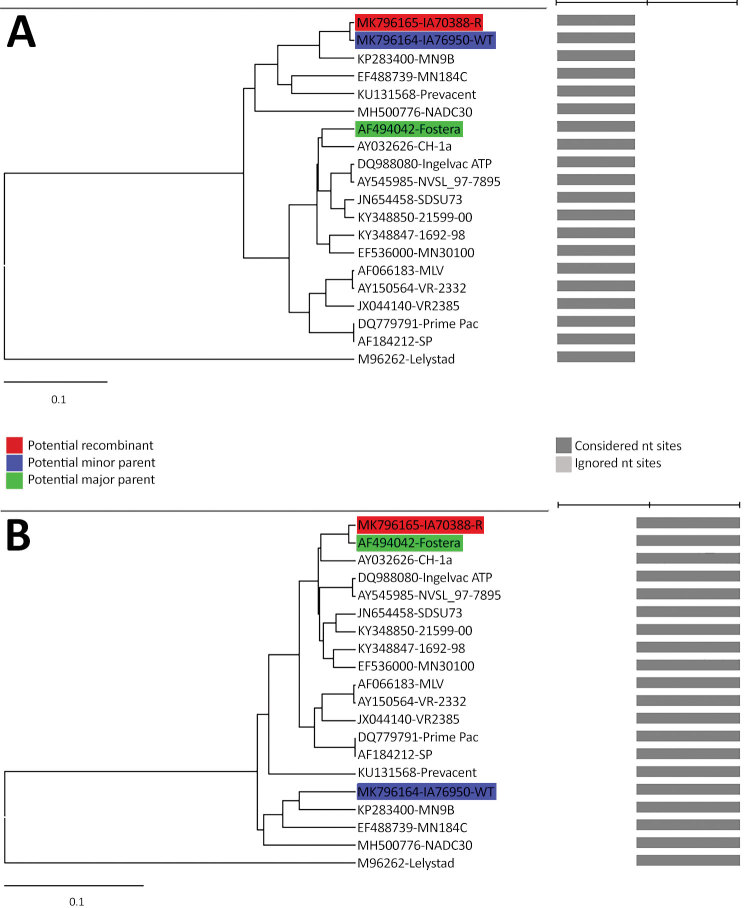
Genome recombination analysis of the IA70388-R strain of porcine reproductive and respiratory syndrome virus, United States, 2018. A) UPGMA of region derived from major parent (1–6742). B) UPGMA of region derived from major parent (6743–15642 nt). Phylogenies of the parent strains were identified using RDP version 4.24 software (http://web.cbio.uct.ac.za/~darren/rdp.html). Red indicates the recombinant (IA70388-R); green indicates the major parent strain (the Fostera vaccine strain); blue indicates the minor parent strain (IA76950-WT). Scale bars indicate nucleotide substitutions per site.

All thus far reported recombinant strains from vaccine and field strains in Europe and Asia were based solely on the bioinformatics prediction, and their wild-type parent strains were only theoretically deduced but not actually identified (*8**–**10*). In this study, we provide solid evidence that a natural recombinant virus evolved from a vaccine strain and a field strain in the United States. The virulence of the recombinant appeared to be reversed, although a pathogenicity study is still needed to confirm. Our study emphasizes the importance of monitoring recombination between vaccine and field strains in swine herds and reiterates the limitations of ORF5-based sequencing for PRRSV characterization, highlighting that full-length genome sequencing is more reliable.

Appendix Additional methods for study of recombination between vaccine and field strains of porcine reproductive and respiratory syndrome virus, United States, 2018.
