# Supplementary material for: Recombination between Vaccine and Field Strains of Porcine Reproductive and Respiratory Syndrome Virus
Source: Emerg Infect Dis. 2019 Dec;25(12):2335–7. doi: 10.3201/eid2512.191111 (PMC6874241; doi:10.3201/eid2512.191111)
Supplement: Appendix — Additional methods for study of recombination between vaccine and field strains of porcine reproductive and respiratory syndrome virus, United States, 2018. [file 19-1111-Techapp-s1.pdf]

Article DOI: https://doi.org/10.3201/eid2512.191111

Recombination between Vaccine and Field Strains of Porcine Reproductive and Respiratory Syndrome Virus

Appendix

Methods

NGS

To further characterize the PRRSV detected by the ORF5 sequencing and Fostera specific PCR, lung samples were subjected to next-generation sequencing (*1*). Specifically, DNA in the extracted DNA/RNA from these samples was removed with RNase-Free DNase Set (Qiagen, Valencia, CA, USA), and reagent residual was then removed from the remaining RNA with Agencourt® RNAClean® XP (Beckman Coulter, Indianapolis, IN, USA) kit according to the kit manual. The library was prepared with NEXTflex Rapid RNA-Seq Kit (Bioo Scientific, Austin, TX, USA) until "step D" according to kit manual with minor modification, and followed with Nextera XT DNA library preparation kit (Illumina, San Diego, CA, USA). Normalized library was sequenced on MiSeq platform (Illumina) with 300-cycle MiSeq Reagent Micro Kit V2 (Illumina).

Bioinformatics Analysis

Raw sequencing data were subjected to data cleaning by removing adapters, trimming low-quality ends, depleting sequences with length <36 nt, and sequencing quality analysis with FastQC (*2*). Taxonomy of cleaned reads was classified using Kraken v0.10.5-β (*3*). Reads of particular/interested viruses were extracted from the kraken classification results as candidate reads of that taxon. Particularly, PRRSV reads were extracted and were de novo assembled with SPAdes (v 3.5.0) as described previously (*4*).

References

1. Chen Q, Wang L, Zheng Y, Zhang J, Guo B, Yoon KJ, et al. Metagenomic analysis of the RNA fraction of the fecal virome indicates high diversity in pigs infected by porcine endemic diarrhea virus in the United States. Virol J. 2018;15:95. PubMed https://doi.org/10.1186/s12985-018-1001-z

2. Zhang J, Zheng Y, Xia XQ, Chen Q, Bade SA, Yoon KJ, et al. High-throughput whole genome sequencing of porcine reproductive and respiratory syndrome virus from cell culture materials and clinical specimens using next-generation sequencing technology. J Vet Diagn Invest. 2017;29:41–50. PubMed https://doi.org/10.1177/1040638716673404

3. Wood DE, Salzberg SL. Kraken: ultrafast metagenomic sequence classification using exact alignments. Genome Biol. 2014;15:R46. PubMed https://doi.org/10.1186/gb-2014-15-3-r46

4. Bankevich A, Nurk S, Antipov D, Gurevich AA, Dvorkin M, Kulikov AS, et al. SPAdes: a new genome assembly algorithm and its applications to single-cell sequencing. J Comput Biol. 2012;19:455–77. PubMed https://doi.org/10.1089/cmb.2012.0021

Appendix Table. Nucleotide identity of IA70388-R compared with IA76950-WT and other representative PRRSV strains.

Genomic region	IA76950-WT	VR-2332	NADC30	MN 9B	SDSU73	MN 184C	CH-1a	NVSL 97–7895	21599–00	1692–98	MN30100	VR 2385	SP	Fostera	Ingelvac MLV	Ingelvac ATP	Prime Pac	Prevacent
Complete genome	**93.0**	86.0	86.6	90.6	86.1	87.5	86.2	86.2	85.6	86.2	84.9	83.5	85.0	88.4	85.9	85.7	85.7	85.5
ORF1a	**96.8**	77.7	84.5	92.5	77.7	87.8	77.2	77.4	77.4	77.3	77.0	74.4	77.2	78.0	78.4	77.3	77.2	84.9
Nsp1α	**99.3**	89.1	90.4	95.6	88.0	90.9	87.6	88.5	88.0	88.5	87.8	89.6	88.5	88.5	89.1	87.8	88.5	88.0
Nsp1β	**99.0**	79.5	81.0	92.0	76.6	85.6	77.2	77.0	76.1	77.2	74.8	80.7	80.9	76.2	80.1	76.7	80.9	80.2
Nsp2	**99.1**	68.7	84.9	94.4	69.0	87.8	68.9	68.4	68.7	68.6	68.3	61.6	67.9	68.1	70.2	68.4	67.9	84.2
Nsp3	**99.3**	83.9	80.9	94.6	82.8	90.6	82.9	82.2	82.3	82.1	82.6	83.5	85.2	82.3	83.8	82.3	85.2	87.1
Nsp4	**99.8**	83.0	83.2	92.6	83.8	89.2	83.3	83.8	83.5	83.7	83.5	84.2	81.7	82.5	83.0	84.0	81.7	87.1
Nsp5	**96.1**	83.5	83.7	91.8	84.7	88.4	83.1	85.3	84.1	83.3	84.3	83.3	84.5	86.3	83.5	85.3	84.5	87.8
Nsp6	87.5	100.	93.8	91.7	93.8	93.8	93.8	93.8	93.8	91.7	89.6	100.0	95.8	**100.0**	100.0	93.8	95.8	93.8
Nsp7a	84.3	94.2	86.8	83.7	93.6	84.0	93.6	94.4	93.5	94.2	94.2	93.7	92.4	**99.8**	94.2	94.5	92.5	83.4
Nsp7b	74.8	89.1	82.4	77.9	93.7	82.5	89.3	90.9	93.0	92.1	91.5	89.7	88.2	**99.7**	90.1	91.2	89.3	81.6
Nsp8	89.6	94.8	88.1	89.6	95.6	89.6	92.6	96.3	95.6	93.3	94.1	95.6	92.6	**100.0**	94.8	96.3	92.6	88.9
ORF1b	86.7	92.8	88.9	87.6	94.9	88.6	95.0	95.4	94.2	94.9	94.4	92.8	92.8	**99.6**	92.9	95.3	92.8	88.9
Nsp9	86.7	93.0	87.8	87.9	95.3	88.6	95.3	95.6	94.7	94.9	94.5	93.4	93.1	**99.7**	93.0	95.4	93.1	87.7
Nsp10	86.7	82.6	87.8	87.7	94.4	89.1	95.3	96.4	93.6	96.0	95.0	91.7	92.6	**99.3**	92.5	96.2	92.6	89.0
Nsp11	87.5	92.4	92.9	87.9	94.8	88.6	94.8	93.8	94.3	93.8	94.2	93.9	92.3	**99.7**	92.6	93.6	92.3	91.3
Nsp12	85.3	93.3	90.7	85.9	94.3	87.7	93.4	94.2	93.7	93.9	92.2	92.9	92.9	**99.8**	93.6	94.3	93.0	90.0
ORF2a	86.6	95.2	87.7	88.6	94.2	88.7	96.1	93.9	93.1	94.9	94.0	94.9	93.1	**99.9**	94.9	93.9	93.1	89.6
ORF2b	86.5	93.7	89.2	90.1	94.6	90.5	96.4	94.6	91.9	95.5	94.1	94.1	93.2	**99.5**	93.2	94.1	93.2	91.4
ORF3	85.1	91.8	84.8	86.4	94.6	88.2	95.9	94.9	94.1	95.0	95.0	92.7	91.0	**99.7**	91.8	94.6	91.0	90.1
ORF4	87.9	92.4	88.5	88.5	97.3	88.8	96.7	95.8	94.9	97.1	96.4	91.5	94.0	**99.8**	92.6	95.5	94.0	91.1
ORF5	84.8	91.6	86.8	85.6	94.2	86.9	96.5	94.2	92.4	94.0	94.4	90.4	92.5	**99.7**	91.2	93.4	92.5	86.4
ORF6	89.5	95.4	89.3	91.4	98.0	92.2	98.1	97.0	95.0	96.4	96.6	95.4	94.3	**98.9**	95.6	96.6	94.3	92.8

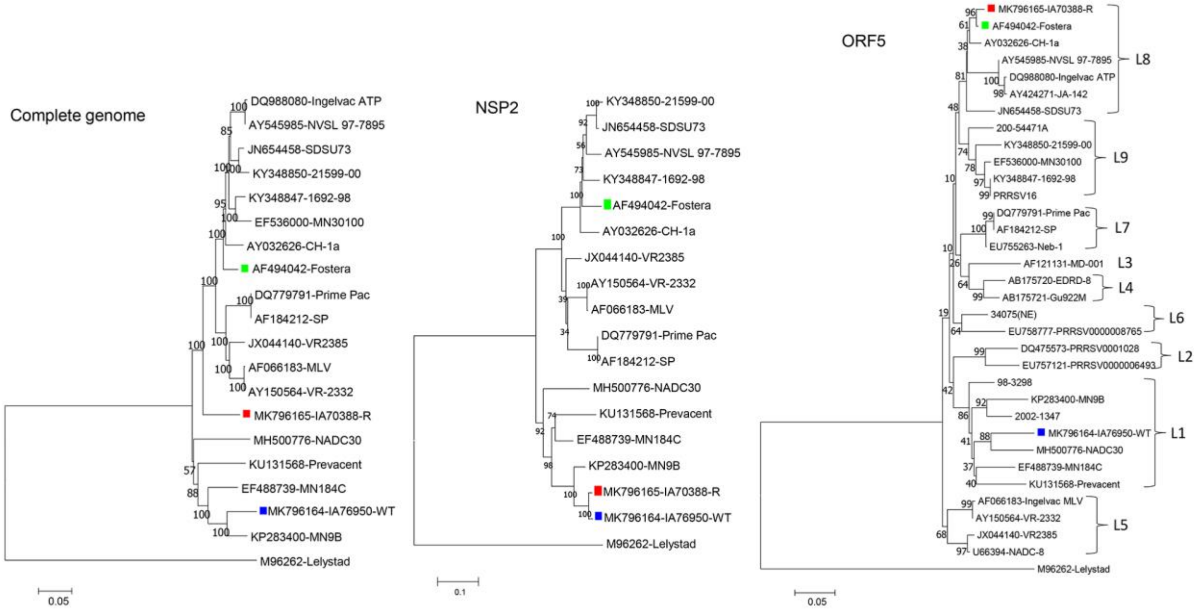

Appendix Figure 1. Phylogenetic analysis of the full-length nucleotide sequence (A), Nsp2 aa sequence (B), and ORF5 nt sequence (C) of PRRSV strains. The phylogenetic trees were constructed by the neighbor-joining method in MEGA 7.0. Bootstrap values from 1,000 replicates are indicated for each node. Each isolate is presented by the GenBank accession number and the isolate name. The strains isolated in the present study, IA76950-WT and IA70388-R, and Fostera vaccine strain are indicated with blue, red, and green square symbols, respectively.

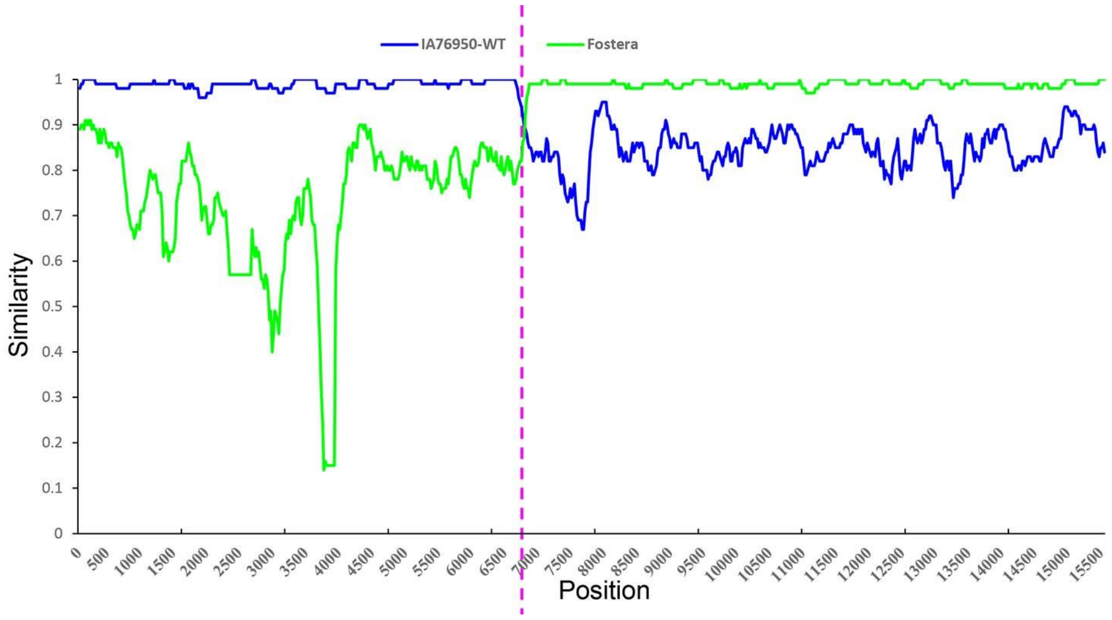

Appendix Figure 2. Similarity plot analysis using the IA70388-R strain of PRRSV as the query sequence against the IA76950-WT strain and the Fostera vaccine strain. A recombination breakpoint is shown with a purple dotted line and the location is underscored at the nucleotide site.
